# Holoprosencephaly in an Egyptian baby with ectrodactyly-ectodermal dysplasia-cleft syndrome: a case report

**DOI:** 10.1186/1752-1947-6-35

**Published:** 2012-01-24

**Authors:** Kotb Abbass Metwalley Kalil, Hekma Saad Fargalley

**Affiliations:** 1 Department of Pediatrics, Faculty of Medicine, Assiut University, Assiut, Egypt

## Abstract

**Introduction:**

Ectrodactyly-ectodermal dysplasia-cleft lip or palate syndrome (OMIM No. 129900) is characterized by the triad of ectrodactyly, ectodermal dysplasia and facial clefting (of the lip and/or palate). Holoprosencephaly denotes a failure in the division of the embryonic forebrain (prosencephalon) into distinct lateral cerebral hemisphere. The association between ectrodactyly-ectodermal dysplasia-cleft lip or palate syndrome and holoprosencephaly is very rare. Here we report holoprosencephaly in an Egyptian infant with ectrodactyly-ectodermal dysplasia-cleft lip or palate syndrome.

**Case presentation:**

An 11-month-old Egyptian female baby was referred to our institution for an evaluation of poor growth; the pregnancy and perinatal history were uneventful. On examination, her growth parameters were below the third centile, she had bilateral ectrodactyly of both hands and feet, dry rough skin, sparse hair of the scalp and operated right cleft lip and cleft palate. Computerized tomography of her brain revealed holoprosencephaly.

**Conclusion:**

The importance of the early diagnosis of this syndrome should be emphasized in order to implement a multidisciplinary approach for proper management of such cases.

## Introduction

Ectrodactyly-ectodermal dysplasia-cleft lip or palate syndrome (EEC syndrome) (OMIM No. 129900) is characterized by the triad of ectrodactyly, ectodermal dysplasia and facial clefting (lip and/or palate). It is a complex, pleiotropic, multiple congenital anomaly or dysplasia in which any of the three cardinal signs can present with variable expression. It may also be associated with many defects not necessarily of ectodermal origin [1]. EEC is inherited as an autosomal dominant trait of low penetrance and variable expressivity. Sporadic cases have also been reported. It was first described by Cockayne in 1936 [2]. The simultaneous presence of these three anomalies is extremely rare, with an estimated incidence of 1.5 per hundred million births [3]. Holoprosencephaly (HPE) represents congenital malformations of the developing forebrain. The combination of EEC syndrome and HPE is very rare, with only 15 cases known to date in the English literature [4]. The true prevalence of EEC syndrome with HPE in Egypt is unknown. A case seen in our institution necessitated a literature review and report.

### Case presentation

**Figure 1:**
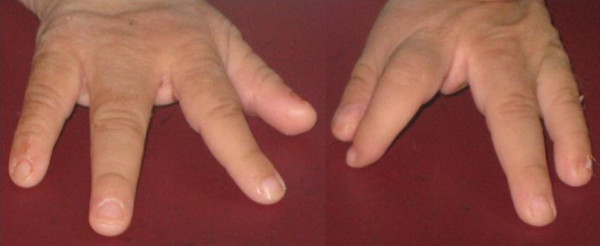
**Ectrodactyly of hands**.

**Figure 2:**
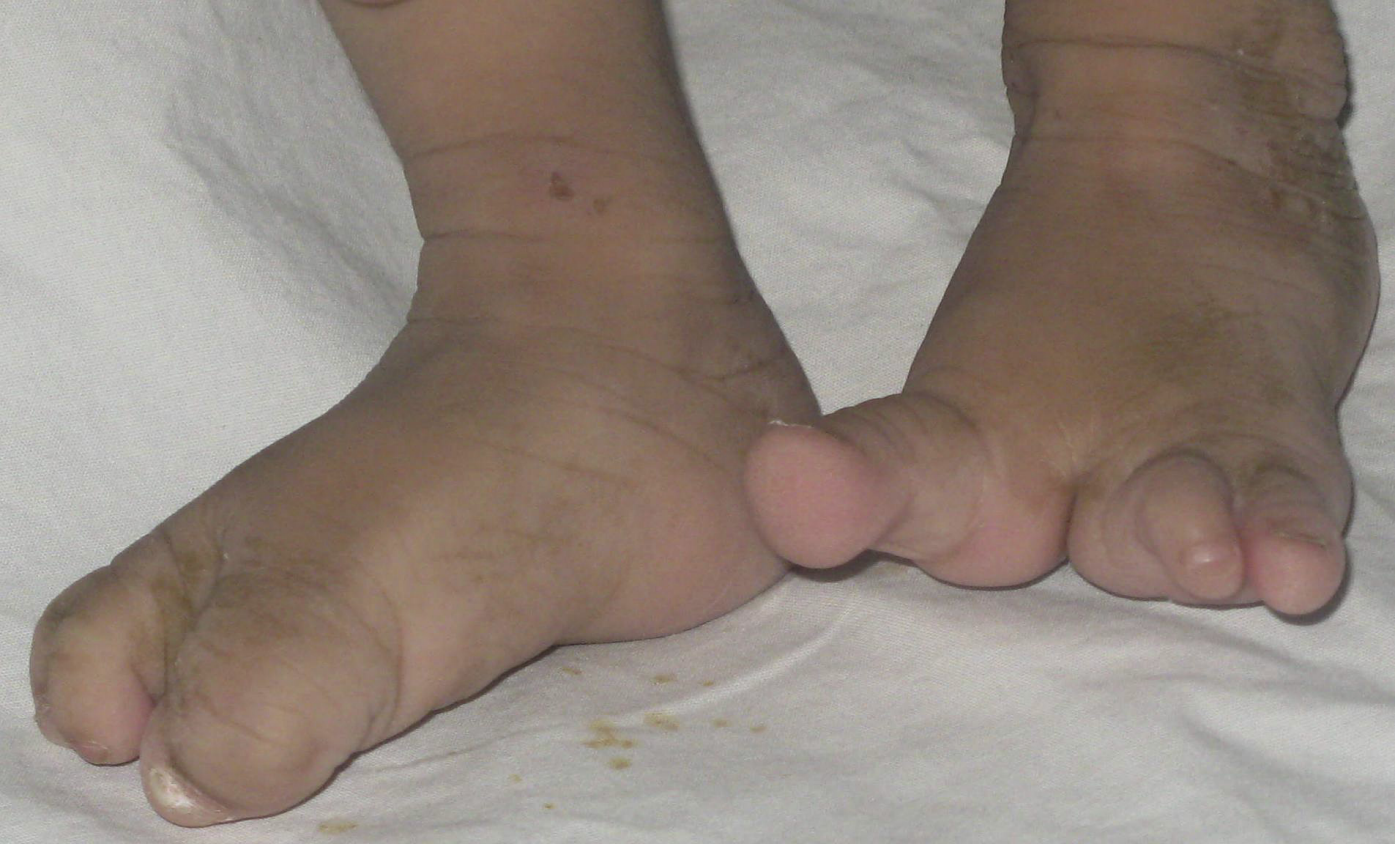
**Ectrodactyly of feet**.

**Figure 3:**
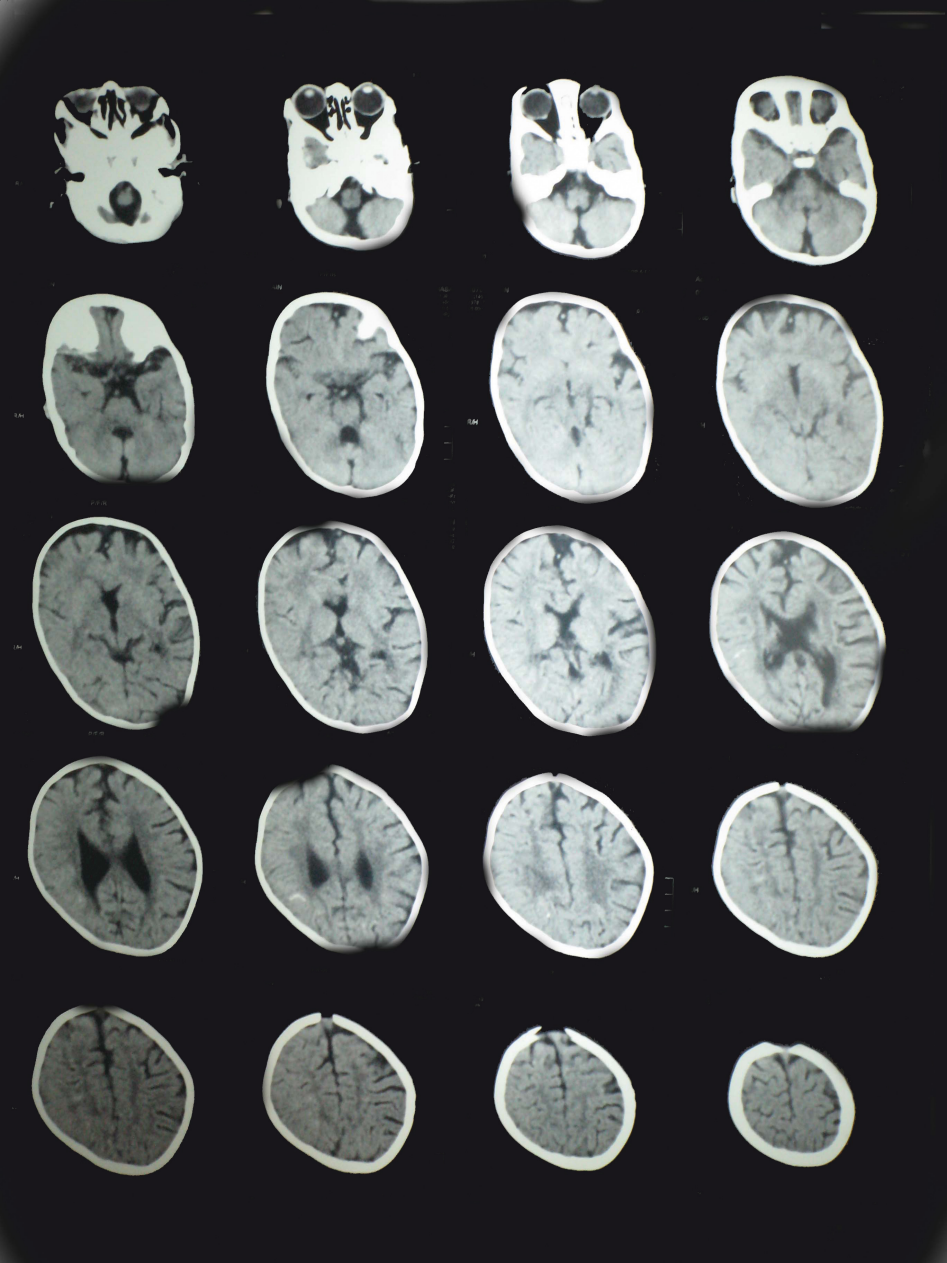
**Computed tomography of her brain showing holoprosencephaly with absent corpus callosum**.

An 11-month-old Egyptian female baby was referred to our institution for an evaluation of poor growth. She was the first child of healthy and unrelated Egyptian parents. The pregnancy was normal and there was no history of hyperthermia, hypertension, diabetes or exposure to toxic, traumatic or infectious agents or radiation. Delivery was through Cesarean section, at term. Her birth weight was 2,900 g (10th centile) and her length was 45 cm (third centile). A right cleft lip-palate and limb anomalies were noted at birth. On examination, her length, weight and head circumference were below the third centile; she had delayed motor and mental development and she had the scar of repair of her right cleft lip, which was done at the age of two months. An examination of her mouth revealed the presence of a cleft palate. Her scalp hair was sparse and hypopigmented and her skin was dry and rough. Both hands showed a classic lobster claw deformity: her middle digit was absent and the remaining four fingers were parted, two on either side, the cleft almost dividing the palm into two (Figure 1). The second and third toes of both her feet were missing and the remaining three toes were divided by a cleft into two parts, the big toe on one side and the other two toes on the other side (Figure 2). Her external genitalia were female. Laboratory tests, including a complete blood count, electrolytes and liver tests and kidney, thyroid, anterior and posterior pituitary functions tests, showed no abnormalities. A chest radiography, abdominal ultrasound, echocardiography, ophthalmologic examination, fundus examination, cerebrospinal fluid pressure and hearing tests were normal. Skeletal X-rays (with the exception of the described limb anomalies) were all normal. Her karyotype was 46, XX and a genetic study demonstrated no mutations in any of the coding regions of TP63. In addition, no chromosomal abnormalities have been identified in her parents. Computed tomography of her brain showed HPE and corpus callosum dysgenesis (Figure 3).

## Discussion

Ectrodactyly refers to a deficiency or absence of one or more of the central digits of the hands and feet. Ectodermal dysplasia involves organs derived from embryonic ectoderm, which can involve both the superficial ectodermal layer as well as the deeper mesoectodermal layer, formed from the neural crest [5]. Other ectodermal anomalies include mild hypohidrosis; coarse, dry hair with hypotrichosis; xerostomia; dystrophic nails; and dental enamel hypoplasia with microdontia. Associated anomalies include blepharophimosis, lacrimal duct anomalies, deafness, choanal atresia and abnormalities of the genitourinary tract. EEC syndrome results from simultaneous ectodermal and mesodermal developmental defects [5]. Although any of the three cardinal signs can present with variable expression and can occur as a separate entity, the combination of all three anomalies appears to be a rare occurrence [6].

HPE is a complex brain malformation affecting both the forebrain and the face. The etiology is heterogeneous: teratogens, chromosomal abnormalities and single gene mutations can be involved [7]. HPE is estimated to occur in one in 10,000 to 20,000 live births [8]. According to severity, HPE is categorized into three forms: a lobar HPE, or complete absence of midline forebrain division resulting in a monoventricle and fused cerebral hemispheres; semilobar HPE, or incomplete forebrain division resulting in partial separation of the cerebral hemispheres; and lobar HPE, or complete ventricular separation with focal areas of incomplete cortical division [9].

Facial anomalies are thought to have a common origin with the intracranial abnormalities and are caused by incomplete cleavage during embryologic development. The association between facial anomalies and HPE has led to the well-known phrase, 'the face predicts the brain'. While this statement is generally true, identical facial features are occasionally recognized in the absence of HPE. Also, facial abnormalities are not invariably present, so that reliance on them will result in false negative diagnoses of HPE [10].

In 1984, Hartsfield *et al. *[11] described the first known case of a child born with HPE and ectrodactyly. Since that time, this combination has been described as comprising a distinct genetic syndrome: HPE, ectrodactyly and bilateral cleft lip and cleft palate syndrome, also known as Hartsfield syndrome (OMIM 300571). While ectrodactyly is a consistent finding, other limb anomalies, such as radial hypoplasia and polydactyly, have been reported in patients with this association, though it is possible that these are etiologically distinct entities [12,13]. Thin hair has been described in patients with HPE-ectrodactyly [14]. Taken together, these data suggest that the etiology of HPE-ectrodactyly may be distinct from that of EEC syndrome. Among patients with HPE and ectrodactyly, the presence of a common phenotype has prompted interest in identifying a unifying cause. To date, however, such causes have remained elusive.

A paucity of reports of EEC syndrome and HPE has prompted us to report this case. Our patient had the triad of ectrodactyly of both hands and feet; ectodermal dysplasia in the form of dry rough skin with sparse, hypopigmented hair; and unilateral right sided cleft lip and cleft palate, in addition to HPE, which fulfills the characteristics features of Hartsfield syndrome.

Our index case had unilateral cleft lip and cleft palate in contrast to Hartsfield syndrome, which describes bilateral cleft lip and cleft palate. This causes some doubt as to whether the incomplete forms reflect a reduced expression of the gene or one or more separate clinical entities.

## Conclusion

The importance of the early diagnosis of this syndrome should be emphasized in order to implement proper management of such cases. Management of cases with HPE in EEC syndrome requires a multidisciplinary approach that includes a dermatologist, neurologist, plastic surgeon, ophthalmologist, pediatric endocrinologist and, if needed, a speech therapist [15].

## Abbreviations

EEC syndrome: ectrodactyly-ectodermal dysplasia-cleft lip or palate syndrome; HPE: holoprosencephaly.

## Consent

Written informed consent was obtained from the patient's parents for publication of this case report and any accompanying images. A copy of the written consent is available for review by the Editor-in-Chief of this journal.

## Competing interests

The authors declare that they have no competing interests.

## Authors' contributions

KA and HS diagnosed, investigated, followed-up and managed the patient and drafted the manuscript. Both authors read and approved the final manuscript.
